# Implementation of the global laboratory leadership programme in Burkina Faso: lessons learned and processes toward sustainability

**DOI:** 10.3389/fpubh.2025.1633359

**Published:** 2025-08-22

**Authors:** Emilie Dama, Abdoulaye Nikiema, Andre Coulibaly, Raoul Karfo, Issaka Yameogo, Fabienne Soudre, Alice Kiba, Charles Sawadogo, Isidore Bonkoungou, Habibata Zerbo, Lila Rahalison, Amanda Balish, Julius Manjengwa, Elie Kabre, Anicet G. Dahourou, Jean Sakande

**Affiliations:** ^1^Centers for Disease Control and Prevention (CDC), Division of Global Health Protection (DGHP), Atlanta, GA, United States; ^2^Integrated Quality Laboratory Services, Lyon, France; ^3^Health Sciences Research and Training Unit, Ouagadougou, Burkina Faso; ^4^One Health Platform, Technical Secretary, Ministry of Health, Ouagadougou, Burkina Faso; ^5^Directorate of Biomedical Laboratory, Ministry of Health, Ouagadougou, Burkina Faso; ^6^Central Reference Laboratory, National Public Health Institute, Ministry of Health, Ouagadougou, Burkina Faso; ^7^National Laboratory for Animal Health, Ministry of Agriculture, Animal Resources and Fisheries, Ouagadougou, Burkina Faso

**Keywords:** GLLP, laboratory, leadership, training, sustainability

## Abstract

**Introduction:**

The Global Laboratory Leadership Programme (GLLP) aims to foster and mentor current and emerging laboratory leaders to build, strengthen, and support national laboratory systems using a One Health approach. Six organizations, including the US Centers for Disease Control and Prevention, developed GLLP to address gaps in leadership and management skills, particularly in low- and middle-income countries. This work highlights lessons from implementing GLLP in Burkina Faso, including the approach to sustainability.

**Methods:**

A participatory implementation model was adopted, engaging key international and national stakeholders across all One Health sectors. From 2021 to 2023, Burkina Faso implemented the full GLLP learning package, which encompasses all nine competencies, projects, and mentoring. Following the completion of the pilot cohort’s training, a sustainability plan was developed, leading to the creation of a University Diploma in Laboratory Leadership and Management (UD-LLM) in 2024, with the training of its first cohort completed in 2025. For both the pilot and the UD-LLM cohorts, a monitoring and evaluation framework was applied throughout the process.

**Results:**

A total of 44 participants from Burkina Faso and Mauritania were trained in both the pilot (18) and the first cohort of the UD-LLM (26). Participants from Burkina Faso (43) were from all One Health sectors and worked in laboratories across 10 institutions (public, private, and military) located in nine of the country’s 13 regions. A total of 117 projects including 10 capstone projects, were completed to address gaps identified at institutional or national levels with support from mentors. A low representation of candidates from the animal and environmental sectors was noted. Overall satisfaction with the training was reported for both cohorts.

**Conclusion:**

Burkina Faso is the first country in the French-speaking African region with a sustained GLLP full package integrated into a university curriculum. The early engagement of country stakeholders across sectors ensured the country’s ownership and a path to sustainability. Maintaining a monitoring and evaluation framework is essential for continued improvement. This work demonstrates how early multisectoral engagement and academic integration of the GLLP can create a sustainable model for laboratory leadership development in resource-limited settings.

## 1 Introduction

Several factors influence the ability of the health system to respond effectively to disease outbreaks, including laboratory capacity, a trained health workforce, and robust surveillance systems (1–3). It has been widely shown that an effective healthcare system relies on highly competent and motivated laboratory professionals who are technically proficient and demonstrate strong leadership and management capabilities (4). Unfortunately, many laboratory leaders lack formal management training or experience in laboratory management and leadership (5, 6) even though such competencies are critical for a sustainable national health laboratory system to effectively ensure disease detection, prevention, and response efforts across disciplines (4, 7, 8). There are no modules related to these competencies in the conventional academic training programs for laboratory personnel (9).

In recent years, laboratory systems have often been neglected in public health priorities (1). However, since 2014, several initiatives have been implemented to strengthen laboratory capacity with the support of African and international partners such as the African Society for Laboratory Medicine, the World Health Organization’s Regional Office for Africa (WHO AFRO), and the United States Centers for Disease Control and Prevention, among others (10). Few of these initiatives focused solely on laboratory leadership and management, while others integrated related components as part of broader efforts.

The University of Washington, USA, in collaboration with the WHO Regional Office for the Eastern Mediterranean, created the Certificate Program in Laboratory Leadership and Management that was successfully implemented in 10 countries in the region. This resulted in the training of 17 participants and 11 mentors from clinical and public health laboratories (11). To improve the diagnostic services offered by the laboratories and their compliance with the Stepwise Laboratory Improvement Process Toward Accreditation (SLIPTA) checklist, Zambia implemented a two-year program to strengthen the leadership and management competencies of laboratory managers and quality assurance officers leading to the accreditation of five laboratories one year after the completion of the program (12). Results from an assessment conducted among the clinical laboratory workforce in Botswana to identify and prioritize continuing professional development training needs revealed that 60% of laboratory scientists and technicians are interested in training on laboratory management, leadership and coaching (13). In response to a series of safety incidents in 2014, the US Centers for Disease Control and Prevention (CDC) launched the Laboratory Leadership Service (LLS), a new career-entry fellowship program that provides training in laboratory safety, quality assurance, and leadership development (14, 15).

Efforts to enhance the laboratory workforce development have also been integrated into existing initiatives, such as the Field Epidemiology Training Program (FETP). In 2004, Kenya was the first to modify the FETP curriculum by introducing a laboratory track (L-Track), transforming it into the Field Epidemiology and Laboratory Training Program (FELTP) (16). This adaptation aimed to more effectively involve laboratory scientists in applied field epidemiology, outbreak response, and disease surveillance, aligning with the revised Integrated Disease Surveillance and Response (IDSR) (17). The success of Kenya’s FELTP motivated the adoption of similar programs across Africa and Central Asia, integrating the L-Track into existing FETPs. By 2011, FELTP programs were implemented in 20 countries, including Burkina Faso. However, by 2016, a study revealed that some countries, such as Kenya, Mozambique, Cameroon, Kazakhstan, and Mali, had discontinued the separate L-Track while Ghana, Georgia, Nigeria, Rwanda, and Tanzania continue to offer it (16). Countries that phased out the L-Track cited challenges such as the absence of a standardized laboratory curriculum, and a lack of sufficient laboratory candidates. To address these shortcomings, a key recommendation of the analysis emphasized the need for tailored strategies to strengthen laboratory management and leadership programs. These strategies should focus specifically on laboratory systems and networks to meet current and future workforce demands in clinical and public health laboratories (1, 16). This recommendation aligns with broader findings indicating that effective public health workforce development programs are discipline-specific, competency-based, and rooted in service learning (5, 18).

To address the gap in specialized training for current and emerging laboratory leaders in leadership and management, the World Health Organization (WHO), the Food and Agriculture Organization of the United Nations (FAO), the World Organization for Animal Health (WOAH), the European Centre for Disease Prevention and Control (ECDC), the US Centers for Disease Control and Prevention (CDC), and the Association of Public Health Laboratories (APHL) partnered to develop the Global Laboratory Leadership Programme (GLLP) (4, 19). The framework of this program adopts the WHO’s definition of competency as “the state of proficiency of a person to perform the required practice activities to the defined standard” (20).

Since GLLP’s launch in 2019, it has been implemented in over 30 countries, and it is currently undergoing global implementation. The process involves several key steps and stakeholders from different sectors (21). As of 2024, 14 countries in the African region are implementing the GLLP at varying levels and utilizing different training formats. Only three countries (Sierra Leone, Tanzania, and Uganda), all English-speaking nations, implemented the full package of this program. The remaining countries, which include six French-speaking nations (Congo, Central African Republic, Chad, Gabon, Guinea, and Mali), trained one cohort so far using a partial format due to COVID-19 and/or the lack of funding. Notably, Uganda is the only country to have started the training of its second cohort (22, 23). Only a few countries have progressed in implementing the program’s long-term/sustained approach. In Africa, partners funding the implementation of GLLP, include the US CDC, European Commission, French government, Global Fund, WHO, and WOAH (24).

In 2021, Burkina Faso pioneered the implementation of the full package of GLLP in the French-speaking African region and is also experimenting with a strategy to ensure its ownership and sustainability within the country. This work highlights lessons learned from the GLLP implementation processes in Burkina Faso, including the approach to sustainability and key considerations to assist other countries and implementers.

## 2 Methodology

For the implementation of the GLLP in Burkina Faso, the Integrated Quality Laboratory Services (IQLS) was tasked by US CDC through a cooperative agreement to provide technical and financial assistance, as well as to play a coordination role by working closely with all stakeholders during all the processes.

### 2.1 Phases of the pilot cohort training of GLLP

#### 2.1.1 Pre-planning phase

The implementation process began with a preparation phase during which several activities were conducted in alignment with the GLLP implementation guide. First, both in-person and online meetings were organized with key stakeholders to present the program and get their buy-in and commitment. The key institutions involved included the Secretariat of the One Health platform, the national laboratory Directorate, the National Public Health Institute (NPHI), and the Health Sciences Research and Training Unit (HSRTU) of the Joseph Ki-Zerbo University (JKZU) of Ouagadougou. Given the multisectoral approach of the program, coordination was placed under the leadership of the One Health platform.

A country readiness assessment was conducted to identify the strengths and weaknesses of national health laboratory systems. This analysis aimed at informing the identification of potential improvement projects and gathering expectations of key stakeholders of the program.

#### 2.1.2 Planning phase

Following this analysis, a detailed work plan for implementing the program was developed along with the Terms of Reference (ToRs) of the GLLP technical working group (GLLP-TWG).

The work plan outlined the program’s objectives, and described the profiles of participants, mentors, and facilitators, as well as the selection process and criteria. It also defined the learning methodologies which included a didactic component, implementation of individual projects and capstone projects. Furthermore, the work plan provided an implementation timeline, detailed the evaluation process, and highlighted the program’s sustainability strategies. On April 2, 2021, the GLLP was officially launched in Burkina Faso by the Minister of Environment, Green Economy, and Climate Change, who was also the Chair of the Technical Steering Committee of the One Health platform.

#### 2.1.3 Implementation phase of the pilot cohort

A GLLP-TWG was established under the leadership of the One Health platform Technical Steering Committee’s Chair. The Chairman and Vice-Chairman of this TWG were, respectively, the Director of the National Livestock Laboratory and the National Director of Medical Biology Laboratories. Other members came from a number of different organizations, including the National Environmental Quality Laboratory (LAQE, Laboratoire d’Analyse de la Qualité de l’Environnement), the National Agency for Environmental, Food, Occupational and Health Product Safety (ANSSEAT, Agence Nationale pour la Sécurité Sanitaire de l’Environnement, de l’Alimentation, du Travail et des Produits de Santé), the Institute for Research in Applied Sciences and Technology (CNRST, Centre National de la Recherche Scientifique et Technologique), the Institute of Environment and Agricultural Research, the Ministry of Higher Education, Scientific Research and Innovation, the Joseph Ki-Zerbo University of Ouagadougou, and partner organizations like the US CDC, WHO, FAO and IQLS. The group met quarterly in ordinary sessions to assess progress in implementation of the program and to provide recommendations.

As part of its mission, the GLLP-TWG oversaw the selection of participants and mentors using predefined criteria. Mentees were then paired with mentors according to their sector, expectations, and learning objectives. A group of facilitators was identified based on their expertise and experience in GLLP competencies. Before sharing the modules with the facilitators for adaptation and customization, the implementing partner translated all the learning materials into French. A working session was then conducted with the facilitators and the GLLP-TWG to review each module.

In-person training sessions were conducted from September 2021 to January 2023 for the participants. Online sessions, each lasting approximately two hours, were organized specifically for the mentors during the same period. Following these sessions, participants designed and implemented individual and capstone projects with support from their mentors. A graduation ceremony was organized, during which participants presented their projects (individual and capstone), discussed their implementation, and shared the outcomes.

Following the successful completion of this first cohort, a review meeting was held to discuss the overall implementation of the program. Feedback from stakeholders including participants, mentors, facilitators, and representatives of the GLLP-TWG as well as the key lessons learned, and the challenges encountered were collected. Key recommendations were made to ensure the program’s long-term sustainability.

The training of the pilot cohort included:

- Didactic sessions for the teaching of all 9 competencies: 1- Laboratory System, 2- Disease Surveillance and Outbreak Investigation, 3- Emergency Preparedness, Response, and Recovery, 4- Biosafety and Biosecurity, 5- Leadership, 6- Management, 7- Communication, 8- Quality Management System, 9- Research
- The development and implementation of improvement of small projects and capstone with support from mentors.
- The community of practice.
- Monitoring and evaluation

### 2.2 Integration of GLLP into a university curriculum (first cohort)

#### 2.2.1 Pre-planning phase

**Figure 1:**
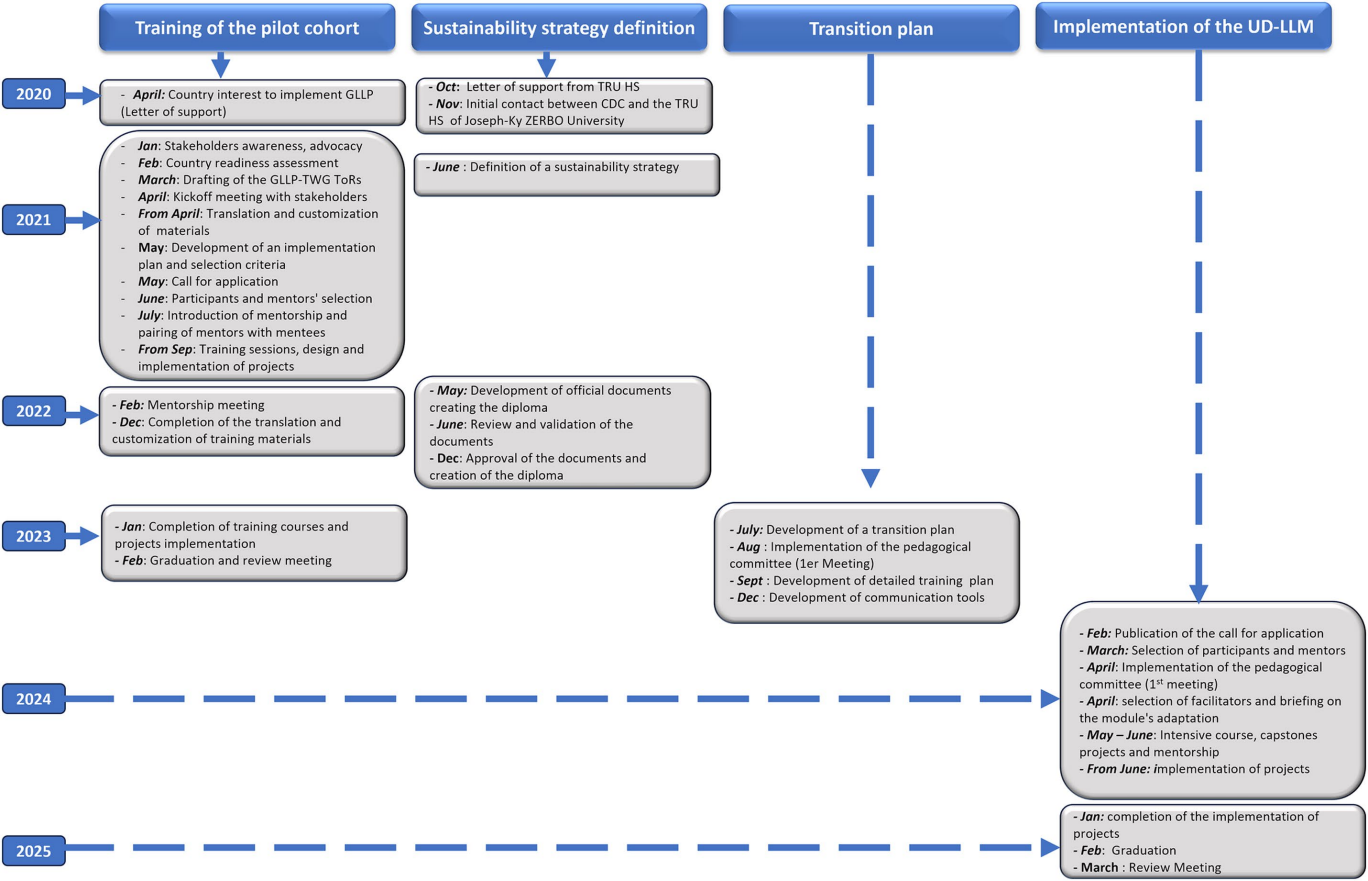
Flowchart detailling a multi-year plan of GLLP implementation and sustainability through integration into University (2020- 2025). GLLP-TWG: global laboratory leadership programme - technical working group. ToRs: terms of reference; TRUHS: training and research in health sciences unit.

The integration of the GLLP modules into the university curriculum had the following key steps, as illustrated in Figure 1. Discussions with Joseph Ki-Zerbo University, the first public university in Burkina Faso located in Ouagadougou, began in February 2020 when the country initiated the implementation of the pilot cohort. The Director of Specialized Training in Laboratory Medicine at Joseph Ki-Zerbo University’s HSRTU expressed the university’s interest in incorporating GLLP modules into its curriculum through a letter sent to the CDC.

#### 2.2.2 Planning phase

During the implementation of the GLLP pilot cohort, the country-initiated discussions to define the strategy it would like to adopt. These discussions led to the suggestion of two complementary strategies. The first consisted of integrating GLLP content as optional or elective modules in an existing diploma, with eligibility limited to human health laboratory specialists only. With this strategy, only the didactic component of the GLLP would be considered. The second option involved creating a specific certificate called “University Diploma” in Laboratory Leadership and Management (UD-LLM), which would be open to all One Health sectors and neighboring countries. The diploma will be envisioned to include all GLLP components, encompassing didactic sessions, projects, mentoring and a community of practice. Stakeholders ultimately validated the second strategy for further implementation. In accordance with the university procedures, required documents were developed and submitted for approval to the university board. These documents include a decree establishing the pedagogical committee, a decree outlining the organization of the training program, a decision appointing the coordinator of the university diploma and a decision appointing the deputy coordinator.

#### 2.2.3 Implementation phase

The training duration is nine months, consisting of five weeks of intensive didactic training, during which the 43 modules covering the nine competencies of GLLP are taught by selected facilitators, followed by six months of project implementation. Before the launch of the UD-LLM, a transition plan incorporating the outcomes from the review meeting was developed. This plan outlined the goals and objectives, desired outcomes, and measurable indicators. It also defined the roles and responsibilities of key stakeholders involved in the transition phase, identified the major steps to be taken, and specified the resources required, including both financial and human resources. An 8-month planning phase occurred from the development of this plan to the publication of the call for applications. Figure 1 describes the entire process, which took about 10 months from the letter of interest to the creation of the diploma.

### 2.3 Monitoring and evaluation of the course and participants

Throughout the training of the pilot cohort, progress was closely monitored at several levels. Quarterly meetings of the GLLP-TWG were held to discuss challenges and provide recommendations. Monthly calls between CDC headquarters and CDC country office SMEs and the implementing partner allowed updating all parties on the implementation process. These meetings were an opportunity to benefit from CDC SMEs technical expertise who also participated in person and virtually in some trainings or meetings. In addition, regular internal meetings with IQLS staff were also organized. Biweekly reports and monthly bulletins were also produced and disseminated to all stakeholders. To evaluate the learning process, pre-and post-tests were administered before and after the delivery of each module. The overall score used to evaluate the participants’ success in the training was calculated by combining the post-test score, which contributed 60%, with the grade for the presentation of improvement projects, which accounted for 40%. The minimum average required to graduate from the program was 75 out of 100.

For the UD-LLM, monitoring and evaluating the intensive didactic courses were emphasized. During the intensive course, daily evaluations were conducted to assess participants’ satisfaction with the content, their understanding, the teaching methods, and the quality of module delivery. In addition, an overall satisfaction questionnaire was completed at the end of the intensive course.

Participants were evaluated right after completion of each GLLP competency, as well as through a final exam covering all competencies, in accordance with university rules. In addition to the final exam for the intensive course, participants presented their improvement and capstone projects before a jury composed of the pedagogical committee members and were formally evaluated by the jury. Participants were required to pass all exams during the didactic course before proceeding with the project implementation. To find out how well the participants did in UD-LLM, the overall score was made up of their grades on the course exams (which were worth 60%) and the presentations of their personal projects (which were worth 40%). The total score was scaled down to 20, and the minimum passing average was 12.

Quarterly meetings of the scientific committee were held, along with monthly meetings between the implementation partners, CDC headquarters, and country offices as part of the monitoring and evaluation process.

At the completion of the entire UD-LLM program, a review meeting was held to collect feedback from stakeholders to improve the training of the next cohorts.

## 3 Results

### 3.1 Training of the GLLP pilot cohort

A total of 54 applications were received and reviewed, including 33 for participants and 21 for mentors. The selected participants were required to be in an active position and have at least a master’s degree, three years of technical work experience, and at least one year of experience managing a team of at least three staff members. The mentors were required to hold a Ph. D. or equivalent, have five years of technical work experience, and possess at least three years of experience managing a team of at least five staff members.

**Figure 2:**
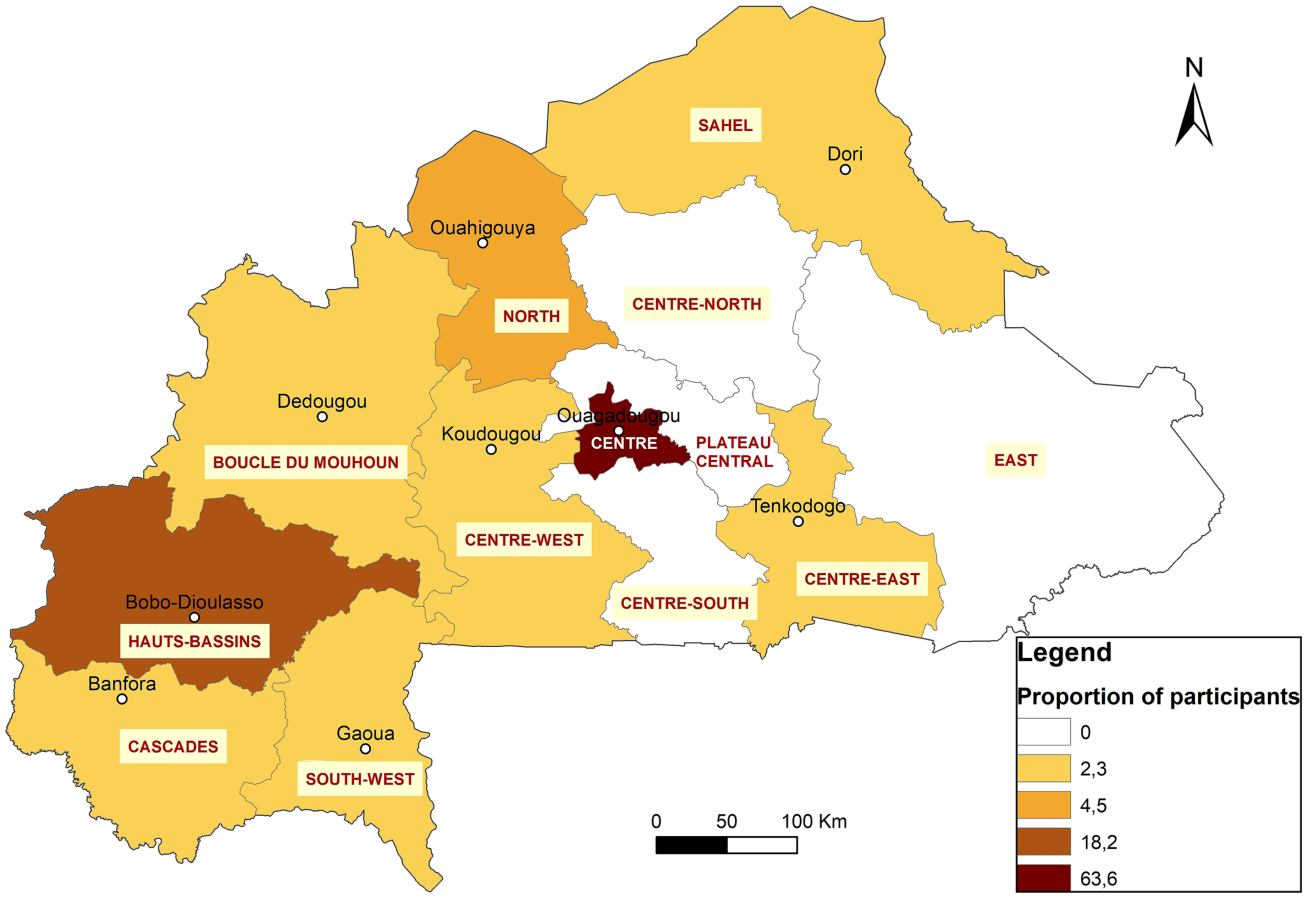
Geographical distribution of the 44 graduates (GLLP and UD-LLM) in Burkina Faso.

After the selection process, 18 participants were enrolled, from laboratories in 9 institutions across 4 regions of the country: Centre, Hauts-Bassins, South-West, and Cascades (Figure 2). The participants included 11 from human health, three from animal health, two from food, and two from environmental sectors. They were paired with nine mentors from the same areas to support them in designing and implementing their projects. Table 1 provides an overview of the participants’ repartition and a list of their affiliated structures.

**Table 1:** Distribution of participants trained in the GLLP pilot phase and in first cohort of UD-LLM.

	GLLP Pilot cohort		UD-LLM first cohort		
Sector	Nb (%)	Participant affiliation structures in Burkina Faso	Nb (%)	Participant affiliation structure	Country
Human health	11 (61%)	National public health institute Teaching hospitals Regional health centers Research centers Military laboratory Specialized health centers	25 (93%)	National Public Health Institute Agency for environmental, food, labor, and health product safety Teaching hospitals Regional health centers Specialized health centers Directorate of Biomedical Laboratories Public health schools and Universities Private laboratory Nouakchott Specialty Hospital	Burkina Faso Mauritania
Animal health	3 (17%)	Animal health reference laboratory	2 (7%)	Animal health reference laboratory Regional animal health laboratory	Burkina Faso
Food	2 (11%)	Agency for environmental, food, labor, and health product safety	0	-	
Environment	2 (11%)	Environmental Quality Analysis Laboratory	0	-	

To cover the 43 modules of the nine GLLP competencies, seven in-person training sessions, each lasting 5 full days were organized for participants, along with 33 online sessions of 2 h each for mentors. 10 facilitators were selected to ensure the delivery of the 43 modules. The average attendance rate was 98% for participants and 54% for mentors. All participants improved their pre-test scores with the overall average score increasing by 21.3% from the pre-test (45.0%) to the post-test (66.3%) (Table 2). The mentors supported the successful implementation of 46 individual and 4 capstone projects related to GLLP competencies (Table 3). All participants successfully completed the training, achieving an average score of 82.20%, within a 10% range.

**Table 2:** Summary of the didactic training sessions of participants of the pilot cohort of GLLP.

Didactic course weeks	GLLP competencies	GLLP modules	Average attendance	Pre and post-test scores difference
Sep 20–24, 2021	Introduction to GLLP and laboratory systems Management	Introduction to the global laboratory leadership programme An introduction to laboratory systems General management Financial management People management Laboratory information systems	100%	+21.3%
Nov 29 − Dec 3, 2021	Leadership	General leadership skills Strategic planning Organizational leadership Critical thinking, problem-solving and decision-making Partnerships and coalition building Ethics in the laboratory	100%	+20.5%
March 21–25, 2022	Quality management system	Introduction to quality management system Process management Documents and records management Equipment and consumables Nonconforming events management Assessments Continual improvement Customer relations	100%	+18.9%
May 10–13, 2022	Biosafety and Biosecurity	Biosafety Biosecurity Shipment of dangerous goods	84%	+31.07%
August 8–12, 2022	Laboratory systems	Laboratory system development: moving forward Model laboratory system overview Policy and legal framework Infrastructure Workforce Information systems Quality management system Biosafety and biosecurity Infectious disease case study	100%	+30.52%
Nov 21–25, 2022	Surveillance and outbreak investigation Emergency preparedness, response, and recovery	Principles of surveillance Outbreak investigation Emergency preparedness Emergency response Emergency recovery	100%	+35.10%
Jan 16–20, 2023	Communication Research and innovation	General communication skills Proposal writing Media relations Risk communication Scientific communication Research and innovation	100%	+45%

**Table 3:** Distribution of projects implemented per competency by the participants.

GLLP competencies	Number of projects implemented (%)	
	GLLP Pilot cohort	UD-LLM first cohort
QMS, general management, leadership	18 (39%)	36 (51%)
Biosafety/Biosecurity	12 (26%)	16 (23%)
Surveillance and emergency management	5 (11%)	4 (6%)
Communication and research	7 (15%)	9 (13%)
Capstone projects	4 (9%)	6 (8%)
Total	46 (100%)	71 (100%)

### 3.2 Training of the first cohort of the UD-LLM

The regional character of the diploma opens it to all applicants from all African Francophone countries across One Health sectors. Due to the widespread dissemination of the opportunity a total of 57 applications were received from Burkina Faso and five other west African countries (Niger, Mauritania, Côte d’Ivoire, Mali, and Togo) (Table 1). The same criteria as the pilot phase was used for the selection of participants and a similar schedule for a didactic course was established over five consecutive weeks, with an average of eight modules taught per week. Thirty-one (25) participants were selected, of which 29 attended the training. Among the attendees, 28 were from 10 institutions located in 7 regions of Burkina Faso (Centre, Hauts-Bassins, North, Sahel, Boucle du Mouhoun, Centre-Est, Centre-West) (Figure 2) and 1 participant was from Nouakchott Specialty Hospital in Mauritania (Table 3). Participants represented both the human and animal health sectors. Additionally, 19 mentors were selected to support the participants. To ensure effective guidance, mentors were paired with mentees at a ratio of 1 mentor to 2 mentees. Sixteen facilitators were selected from various organizations, including the Directorate of Biomedical Laboratory, the epidemiological surveillance department, the Health Emergency Response Operations Center (CORUS - Centre des Opérations de Réponse aux Urgences Sanitaires), university and research institutions, the One Health platform, and international organizations such as WHO, CDC (headquarters and country office), Jhpiego, and IQLS. A total of 65 individual projects and 6 capstones projects related to the GLLP competencies were implemented (Table 3) and outcomes were presented during the graduation ceremony.

Two participants failed because they missed training sessions for certain competencies and could not complete all the required exams during the intensive course, which was necessary for the university to validate the program.

**Figure 3:**
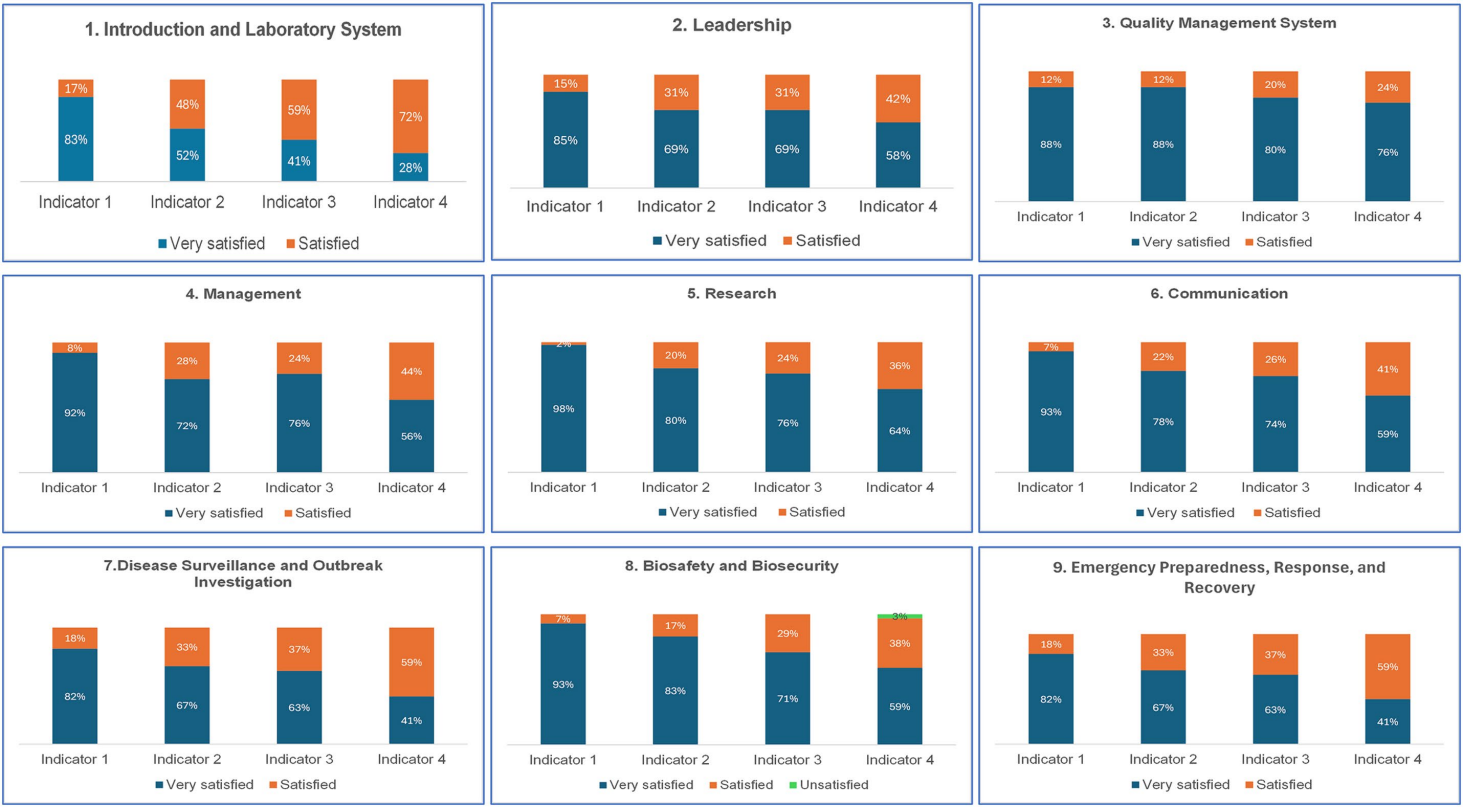
Results from the M&E of the intensive course of the training of the first cohort of the UD-LLM ***Indicator 1***: Relevance of the subject or cases studied and course content; ***Indicator 2***: The facilitator’s mastery of the subject; ***Indicator 3***: Teaching methods and quality of the facilitator’s delivery; ***Indicator 4***: Achieving objectives1.

The evaluation scores by indicators and competencies, as illustrated in Figure 3, revealed that overall, participants were very satisfied (68.58%) or satisfied (25.78%) with the didactic course. However, regarding Biosafety/Biosecurity competency, 3% of participants were unsatisfied.

After the final assessment, 26 out of the 27 anticipated candidates passed the evaluation, while one failed (did not manage to complete and present projects due to occupational commitments abroad). The success rate was 96.3%. The average score of all participants was 15.45 out of 20, with scores ranging from 12.86 to 17.6 out of 20.

## 4 Discussion

Burkina Faso is the first French-speaking African country to successfully implement the full GLLP program. Additionally, the country has distinguished itself as one of the most advanced countries in Africa by designing and implementing a strategy to ensure the sustainability of this training program, as evidenced by its recognition as a University Diploma (UD). This initiative has successfully trained the pilot cohort, marking a significant step in the development of laboratory leadership and management skills in the region. This success was driven by the country’s strong commitment, the expertise of the implementation partner, and the substantial support of GLLP stakeholders, including subject matter experts (SMEs) from CDC. Drawing from these experiences, Burkina Faso is now well-positioned to share lessons learned that can contribute to improving GLLP tools and supporting other countries interested in adopting and successfully implementing this transformative program.

Several countries, particularly in Africa, are interested in the GLLP, where leadership capabilities in the global health workforce, including the laboratory field, are needed to ensure quality healthcare services and an effective response to public health emergencies (26, 27). Implementation planning must incorporate a long-term vision to effectively address the country’s needs. Studies have shown that the sustainability of training programs depends on early integration into national systems and alignment with local priorities (28, 29). The example of Burkina Faso illustrates a proactive approach that led to a successful transition to sustainability without long interruptions. From the earliest stages of the pilot cohort’s implementation, strategic discussions were initiated to define the next steps after this initial phase, ensuring the continued training of laboratory managers.

A systematic review of leadership development programs for healthcare professionals in low- and middle-income countries revealed that these programs predominantly exist in Anglophone countries in Africa (30). By establishing this regional UD-LLM, Burkina Faso enables Francophone countries to benefit from a training program designed to strengthen the leadership and management skills of public health staff in the laboratory field. The consistent attendance and observed learning progression noticed reflect both the relevance of the topics and strong engagement and institutional support for participants to complete the full training. Evaluation of the intensive course for the UD-LLM highlighted a high overall satisfaction among participants regarding the didactic course. This satisfaction can be attributed to the strategic selection of facilitators, chosen for their expertise, experience, and ability to deliver effective adult learning. The diverse backgrounds of the facilitators enhanced the training by providing participants with multidisciplinary perspectives and practical insights on each module. Engaging facilitators with both technical expertise and teaching skills ensures an effective transfer of relevant and applicable knowledge and interactive courses. This method fits with the ideas put forward by Winters et al. (31), which encourage, among other things, that different levels of adult development be taken into account and that different points of view be included in health programs at the college level in order to improve the environment for leadership development. However, to maintain and improve the quality of this diploma, modules should be contextualized with recent examples and case studies that reflect the country or region’s current realities. This could include for instance, experience-sharing sessions from nationally or internationally accredited laboratories, national or regional management of recent outbreaks, tabletop exercises simulating public health emergencies and laboratory response, real-life examples of laboratory leadership in crises, including challenges and lessons learned.

The same selection criteria were used to choose participants and mentors for the pilot cohort as for the first cohort of the UD-LLM. In both cohorts, we observed low representation from the animal health and environmental health sectors, which ranged from 7 to 17%. This disparity can be attributed to the limited number of eligible candidates in these sectors compared to human health, along with their involvement in multiple programs, which consequently limits their availability. Initially, the 31 selected participants included two from the environmental sector. They were unfortunately unable to participate in the course due to other priority activities they had been involved in at the last minute. These observations suggest proactive planning and coordination with environmental and animal health institutions to better align training schedules with sector availability.

The two implemented cohorts trained 44 laboratory staff across nine out of the 13 regions in Burkina Faso. A significant proportion of graduates are concentrated in the capital, Ouagadougou (63%), and in Bobo-Dioulasso (18%), the country’s second-largest city. In fact, these two cities are home to the main reference laboratories that study important human diseases like meningitis, measles, HIV, viral hemorrhagic fevers, tuberculosis, respiratory diseases, rotavirus, papillomavirus, and mycobacteria. A higher number of applications were received from these laboratories that played key roles in epidemiological surveillance as well as in confirming and responding to disease outbreaks and unusual events. Notably, the program included participants from a private laboratory and a military camp laboratory. Given Burkina Faso’s current security challenges, military camp laboratories are crucial in addressing the needs of individuals affected by conflict-related injuries. For instance, managing mass war casualties or responding to an epidemic in a camp requires leadership skills taught in GLLP modules.

On average, each participant from the two cohorts implemented four projects during the training period, with at least one of them directly related to leadership or management. Although other projects did not explicitly focus on leadership and management competencies, the knowledge acquired from the training modules related to these competencies significantly contributed to their successful implementation. Most of these projects involved implementing changes and improvements to align with national or international guidance or requirements to address identified gaps. Therefore, their execution required strategic, knowledge-oriented, value-based, supportive, participatory, and communicative actions, which are part of leadership characteristics as defined by Friman (25). It is well documented that contemporary challenges driven by the rapid pace of change and innovation in the public health sector highlight the evolving role of leaders in health institutions, including laboratories. This situation justifies the critical need to equip these leaders with the skills and competencies necessary for swift adaptation and effective management of public health events (32).

Results show a limited representation of regional laboratories. Given that these laboratories are often on the front line of confirming and managing community health events, greater effort is needed to strengthen their capacities, as these facilities are pivotal in early detection and outbreak response (33). Recent studies on the implementation of the 7–1-7 approach in 41 public health threats in 5 African countries indicate that 61% of bottlenecks were related to detection, with the most frequently observed issue being low awareness or clinical suspicion among health workers (29%), followed by delays in laboratory confirmation (10%) (34). In alignment with the One Health approach, Burkina Faso has committed to enhancing the skills of laboratory managers across human, animal, and environmental health sectors, both at the national and sub-national levels. To translate this vision into action, a budget line was included in national grants, such as the Pandemic Fund and C19RM, to provide scholarships for the DU-LLM. This strategic investment fits with the idea that improving health leadership is a system-wide change that needs help at the individual, team, and system levels. As a result, it should be a part of larger efforts to improve the health system (35–37). For long-term sustainability, DU-LLM program is actively promoted at both national and regional levels so that institutions and ministries integrate this training into their workforce development strategies and allocate funding for their staff to participate.

In addition to the limitations associated with the low representativeness of participants from the animal health and environmental sectors, another limitation of this study is the lack of information on the impact of the pilot cohort two years after training. Although no formal evaluation has yet been conducted, several participant-led projects have contributed to addressing institutional and national-level gaps. A formal assessment of the impact of this training on the use of the knowledge and skills acquired during training, on the participants’ home institutions, on the national health system, and on their professional development will be important to better adapt training for future cohorts. The evaluation’s results could also inform recommendations for optimizing the utilization of this skilled workforce.

While Burkina Faso is the first French-speaking African country to implement the full GLLP package and institutionalize it through a university diploma, experiences from other countries offer valuable perspectives on diverse implementation models. In East Africa, Uganda’s national GLLP program was led by the Uganda National Institute of Public Health in collaboration with Makerere University and Baylor University. This implementation was coordinated with the Field Epidemiology Training Program (FETP), allowing for efficient sharing of financial, technical, and human resources. Similarly, Tanzania and Sierra Leone successfully implemented the full GLLP package but have not yet formalized it through academic pathways. In Central Africa, a regional approach was adopted by WHO AFRO to deliver GLLP training in Congo, Gabon, Central African Republic, Democratic Republic of Congo, and Chad. Furthermore, in Southern Africa, the East, Central and Southern Africa Health Community (ECSA-HC), in collaboration with the Ugandan supranational TB reference laboratory, supported national GLLP programs in Malawi and Zambia, with certification delivered by the ECSA College of Health Sciences. These experiences underline the adaptability of the GLLP model to various national and regional contexts and highlight Burkina Faso’s unique contribution in pioneering a university-based, multisectoral, and sustainable approach to laboratory leadership development.

## 5 Challenges and lessons learned

The implementation and institutionalization of the GLLP in Burkina Faso faced several challenges. However, these were largely addressed through guidance and technical assistance from CDC and GLLP secretariat, as well as the expertise of the implementing partner. The various monitoring and consultation frameworks established (such as GLLP-TGP, UD-LLM scientific committee, regular meetings, and good practice committees via WhatsApp groups) also played a crucial role. The main difficulties encountered are the following:

- Translation of training modules into French: translating training materials from English to French was time-consuming for implementing partners due to the number of modules and the technical nature of their content. This limited the facilitators’ ability to contextualize and adapt module contents effectively before training sessions during the pilot phase.
- Financial support for capstone projects: group project objectives usually extend beyond individual institutions, necessitating financial support for successful implementation. The lack of funding hindered some projects from fully achieving their intended outcomes.
- Implementation of projects: feedback from the training of the pilot phase highlighted that participants were not given enough time to implement their projects effectively. It is critical to define in advance the number of projects required for each participant. Additionally, some participants reported a lack of support from their institutional leadership during project implementation. Furthermore, not all mentors held regular meetings or provided adequate follow-up with their mentees. These circumstances hampered the implementation of some projects.

Key lessons learned for other countries preparing for GLLP implementation:

- Integration of continuity and sustainability: as countries prepare for implementation, they must consider long-term goals for the program right from the outset. Training a single cohort of laboratory leaders is typically insufficient to address existing gaps; therefore, strategies should be devised to ensure continuous and sustainable training that meets national needs. Involving partners early in these discussions allows them to anticipate the financial support requirements in their budgeting processes.
- Implementing partner based in the country: For an initial implementation of the program, it is important to have a partner with strong expertise in laboratory field and based in the country, with a deep knowledge of the country context and its health and education systems, with established and recognized connections with stakeholders.
- Country leadership and engagement: the program should be led by an institution capable of collaborating with multiple partners across various stakeholders within the One Health sector. All stakeholders should be integrated from the beginning, starting with representation from all GLLP partners present in the country and close contact should be maintained with GLLP partners for ongoing support and guidance.
- Equitable selection criteria: to improve equity in participation among different sectors within One Health, selection criteria should be adapted to ensure adequate representation from each sector.
- Financial support for group projects: anticipating a budget line in the work plan specifically for supporting group projects is vital to ensure their successful implementation and achieve the expected results.
- Contextualization of training modules: GLLP modules are generic and need adaptation with examples and case studies that reflect the specific context of the country or region. When selecting facilitators, their ability to contextualize the content effectively should be considered.
- Monitoring and evaluation framework: clear indicators must be defined to effectively monitor the program progress. A technical working group or steering committee should be established to hold regular meetings focused on discussing progress and making recommendations for addressing issues. Additionally, conducting review meetings after each cohort will facilitate feedback collection that can contribute to sustaining and improving program quality.
- Integration of the program into the country’s global health systems: countries should integrate the program into their global health strategies, particularly concerning health workforce development. A system must be established to ensure ongoing training for laboratory managers in leadership and management roles. Furthermore, career advancement opportunities should be offered to retain these professionals within the system.

## 6 Conclusion

Setting up the GLLP in Burkina Faso as a pioneer in the French-speaking African region has proven to be a successful initiative. The creation of a regional university diploma, accessible to professionals from human, animal and environmental sectors related to One Health, marks a significant step forward. This program presents a valuable opportunity to train current and emerging laboratory leaders in leadership and management across the entire African region. Despite facing challenges, the successful implementation of the program was made possible through the unwavering commitment of Burkina Faso and its stakeholders. It is crucial to continue efforts to maintain and enhance the quality of the diploma course so that it qualifies for formal certification in the future. Moreover, health systems in the region must not only promote the training of laboratory personnel in leadership and management to address existing gaps but also create frameworks and opportunities that allow graduates to apply their skills. This will enable them to make meaningful contributions to improving public health. To ensure the long-term impact and effectiveness of the GLLP, it is essential to establish a robust mechanism for monitoring and evaluating the program’s implementation and its outcomes, both for the beneficiaries and the broader health system. The country has initiated a survey to assess the impact of this program at participant, institution and national health system levels. The survey outcomes will be published once available. Building on its experience, Burkina Faso has gained valuable lessons and best practices that can guide other countries toward successfully implementing similar programs.

## Data Availability

The raw data supporting the conclusions of this article will be made available by the authors, without undue reservation.

## Glossary

ANSSEAT: Agence nationale pour la Sécurité sanitaire de l’Environnement, de l’Alimentation, du Travail et des Produits de Santé (National Agency for Environmental, Food, Occupational and Health Product Safety)
APHL: Association of Public Health Laboratories
CDC: Centers for Disease Control and Prevention
CNRST: Centre National de la Recherche Scientifique et Technologique (Institute for Research in Applied Sciences and Technology)
CORUS: Centre des Opérations de Réponse aux Urgences Sanitaires (Health Emergency Response Operations Center)
ECDC: European Centre for Disease Prevention and Control
FAO: Food and Agriculture Organization
FELTP: Field Epidemiology and Laboratory Training Program
FETP: Field Epidemiology Training Program
GLLP: Global Laboratory Leadership Programme
HSRTU: Health Sciences Research and Training Unit
IDSR: Integrated Disease Surveillance and Response
IQLS: the Integrated Quality Laboratory Services
LAQE: Laboratoire d’Analyse de la Qualité de l’Environnement (National Environmental Quality Laboratory)
LLS: Laboratory Leadership Service
NPHI: National Public Health Institute
SLIPTA: Stepwise Laboratory Improvement Process Toward Accreditation
TWG: Technical Working Group
UD-LLM: University Diploma in Laboratory Leadership and Management
WHO: World Health Organization
WOAH: World Organization for Animal Health
